# The Effect of Tuberculin and of Glycerin-extract from Tuberculous Organs

**Published:** 1898-05

**Authors:** 


### ABSTRACTS FROM FOREIGN JOURNALS.

### GERMAN.

Under the Direction of J. Preston Hoskins,
PRINCETON, N. J.

The Effect of Tuberculin and of Glycerin-extract from Tuberculous Organs. (By Garino.) Koch has shown that healthy porpoises can stand 2 g. of tuberculin subcutaneously administered, without visible suffering, while tuberculous ones will die within six to thirty hours after the injection of 0.5 g. of tuberculin. If death occurs before the sixth or after the thirtieth hour, then not the tuberculin, but intercurrent infectious disease (pneumonia, malignant œdema) are demonstrably the cause of death.

Porpoises which have died in consequence of tuberculin show acute inflammation at the point of injection, which becomes tinged with a pronounced violet color; the lymphatic glands of the region are likewise inflamed. The liver and spleen, beside tuberculous changes, show on the surface dark-red, pointed or lentil-shaped spots, which, under the microscope, prove to be enormously distended capillaries which lie around the tubercle. These capillaries are surcharged with red blood-corpuscles, so that circulation seems to be hindered in places. Such spots are found on the lungs, but not constantly. This condition is typical of the tuberculin effect.

From the tuberculous lung of a cow Garino carefully removed 1500 g. of tubercles and extracted them in a 5 per cent. solution of glycerin-water in a thermostat at 100°—two hours the first day, and on the second and third day one hour each. This filtration, in which there were no more living germs, was then steamed down to one-tenth in a water-bath. Three c.cm. of this latter filtration injected subcutaneously into some healthy porpoises caused an abscess which opened speedily and showed little sign of healing. The general condition of the subjects was not disturbed. They even gained in weight.

Tuberculous porpoises died after an injection of 3 g. and more of the extract, but not within six to thirty hours, as in Koch's experiments, so that death was not to be attributed to the effect of tuberculin. The post-mortem examination showed no result pointing that way.

Garino concludes from these experiments that the effect of tuberculin is caused through matter which is formed and multiplied in the bouillon cultures, but which in the living organism is formed only accidentally, or not at all, or which is quickly gotten rid of.—*Deutsche Thierärztliche Wochenschrift.*

